# Posttraumatic Progressive Vertebral Hemangioma Induced by a Fracture

**DOI:** 10.1155/2017/8280678

**Published:** 2017-06-20

**Authors:** Kaya Kilic, Emre Unal, Zafer Orkun Toktas, Fugen Vardar Aker, Akın Akakın, Türker Kilic

**Affiliations:** ^1^Department of Neurosurgery, Bahçeşehir University Faculty of Medicine, Istanbul, Turkey; ^2^Department of Pathology, Haydarpaşa Numune Training and Research Hospital, Istanbul, Turkey

## Abstract

The authors present an extremely rare case of an aggressive and progressive vertebral capillary hemangioma of the lumbar spine secondary to a trauma. A 40-year-old man who complained of back and leg pain due to a hemangioma of L1 that had begun a year after the fracture of the same vertebra was subsequently operated on. Due to the profuse bleeding, only a subtotal removal was possible. Histopathological diagnosis of the lesion revealed a capillary hemangioma. Postoperative control MRI taken at eight months showed that the lesion and destruction of the L1 vertebra were progressive. A second embolization procedure was performed and this time the hemangioma was totally removed via an anterior approach and corpectomy. Fusion was achieved by Th12-L2 graft and plaque. In the fourteenth year of follow-up, he was symptom-free and radiologically clear of this lesion. We propose that progressive hemangioma is extremely rare and that its cure is possible by total surgical removal of the lesion. This case is the second extradural capillary hemangioma secondary to spinal trauma ever to have been documented in English literature. The emergence of a hemangioma in a fractured vertebra suggests that its pathogenesis can be related to the deviation of the angiogenetic pathways from the normal healing process.

## 1. Clinical Presentation

**Figure 1:**
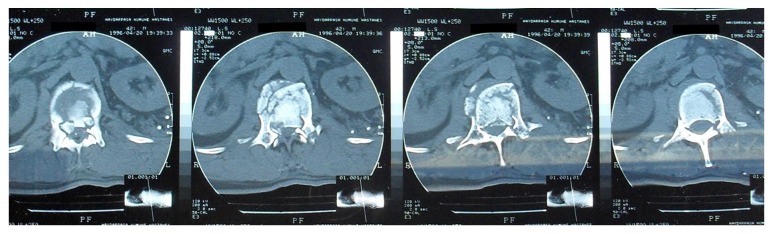
CT: fracture of both pedicles, left transverse process, and anterior and middle column of L1. There is absolutely no hemangioma or any other spinal soft tissue lesions at the left pedicle, left transverse process, and lamina.

**Figure 2:**
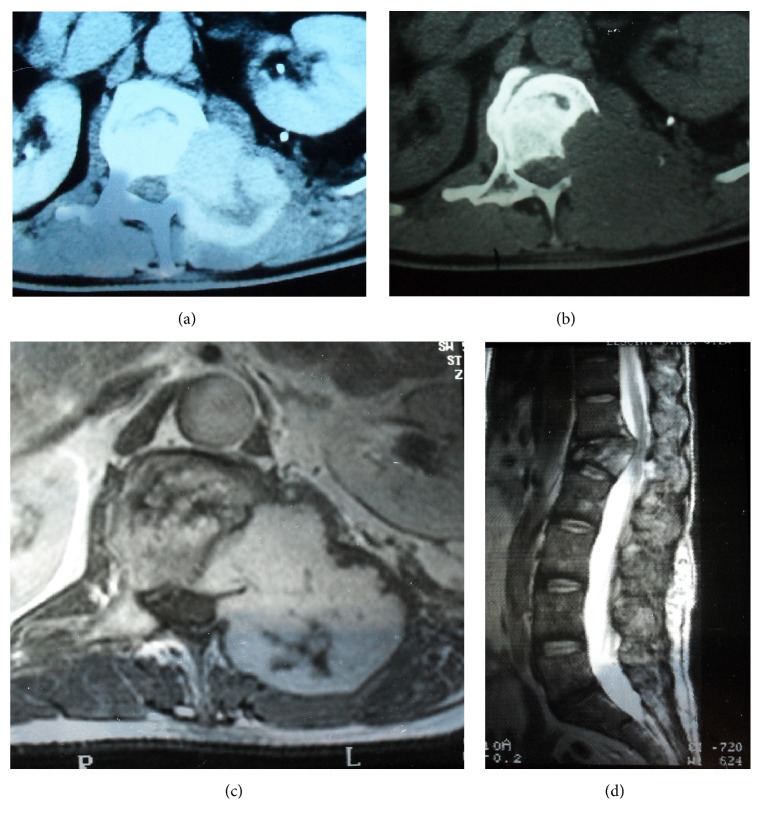
(a) CT: enhancing mass that has destroyed the left half of the body, left pedicle, and left transverse process of the L1 vertebra. (b) CT: bone window, partial destruction of the L1 body, absence of the left pedicle, and left transverse process. (c) MRI, axial T_1_WI, with gadolinium: mass of L1 vertebra destroying the body and compressing the paraspinal soft tissue. (d) MRI, sagittal T_2_WI: the hemangioma invading the spinal canal.

**Figure 3:**
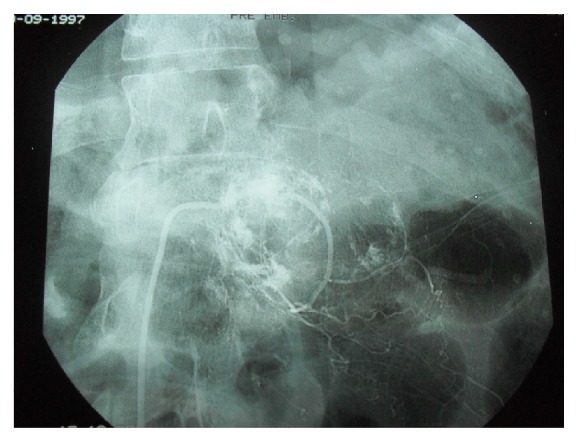
Embolization of the left L1 feeder.

**Figure 4:**
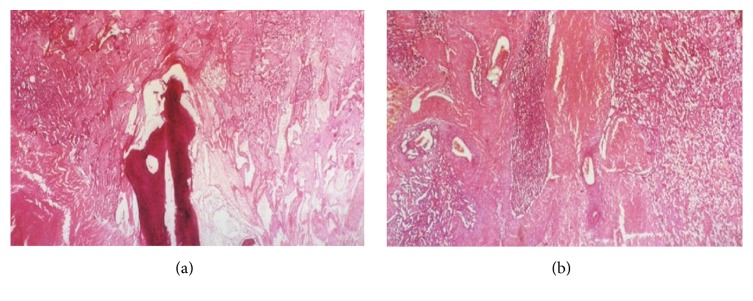
(a) (HE ×10) Vascular formations between osseous and collagenous structures, angiomatosis, and papillary endothelial hyperplasia. (b) (HE ×10) Angiomatosis with vessels of various sizes involving muscle, fat, and bone tissue. A lesion consisted of a collection of large venous and capillary sized vessels scattered haphazardly throughout the soft tissue, striated muscle, and spongious bone. There are also thrombi in formation in some vessels showing papillary endothelial hyperplasia.

A 40-year-old man had a car accident in 1996 which caused the burst fracture of L1 as diagnosed on a spinal CT scan and fracture of left femur diaphysis. This examination revealed absolutely no hemangioma or any other spinal soft tissue lesions (Figure 1). The L1 fracture was treated conservatively with bed rest and bracing. One year after the accident, the patient complained of progressive pain in his back and left leg. MRI and CT scans showed an enhancing tumoral mass that had destroyed one-third of the left L1 vertebra and had expanded to the dural sac, the left L1 root, and the paravertebral muscles (Figures 2(a), 2(b), 2(c), and 2(d)). Surgical resection was planned and preoperative embolization which had to be suboptimal due to technical difficulties was performed (Figure 3). During surgery, severe hemorrhage led to only subtotal removal. The histopathological examination revealed vascular formations between osseous and collagenous structures, angiomatosis, and papillary endothelial hyperplasia conclusive of a capillary hemangioma (Figures 4(a) and 4(b)).

**Figure 5:**
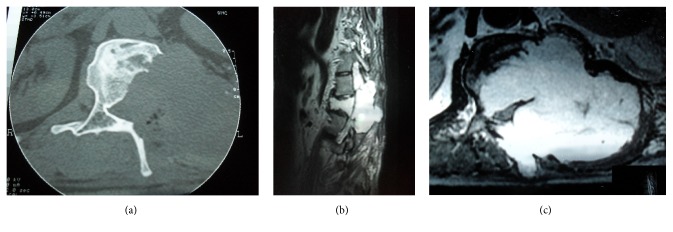
(a) CT: the destruction of the L1 vertebra, compared to Figure 2(b), has obviously become more important. (b) MRI, sagittal T_2_WI: the kyphotic deformity is evidently more prominent. (c) MRI, axial T_1_WI, with gadolinium: the dimensions of the hemangioma have clearly increased; the destruction of the L1 vertebra has obviously become more important.

**Figure 6:**
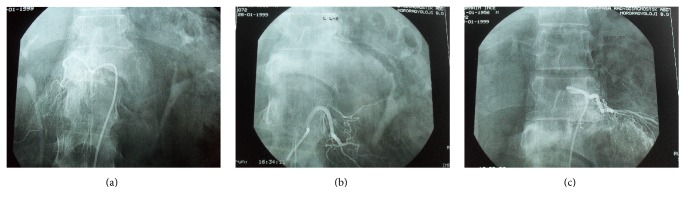
Successful embolization and obstruction of the right L1, left L2, and left Th12 feeders.

**Figure 7:**
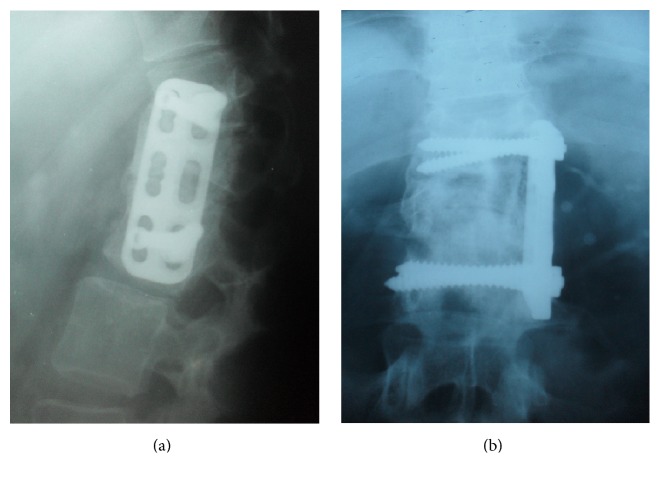
Arthrodesis of Th12-L2 vertebrae by iliac graft and plaque after the corpectomy of L1 by an anterolateral approach.

**Figure 8:**
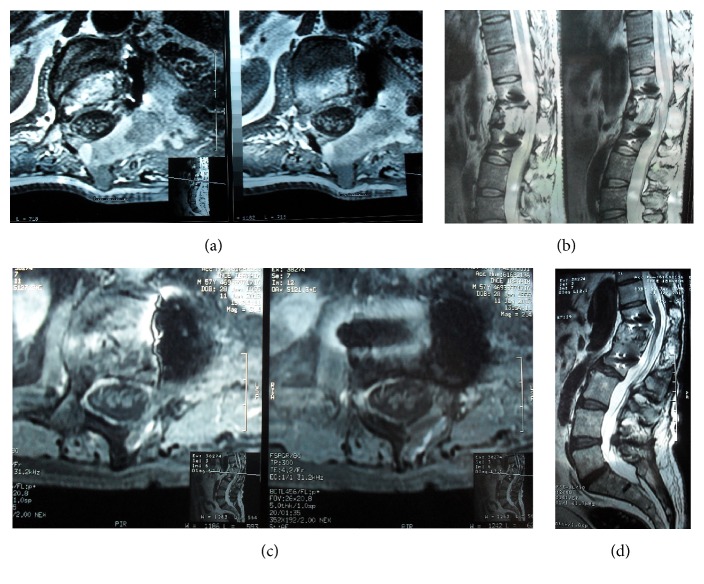
(a, b) MRI, axial T_1_WI and sagittal T_2_WI: the hemangioma is totally removed and the spinal cord is decompressed. (c, d) At the sixteenth year follow-up MRI, axial T_1_WI with gadolinium and sagittal T_2_WI show no recurrence.

**Figure 9:**
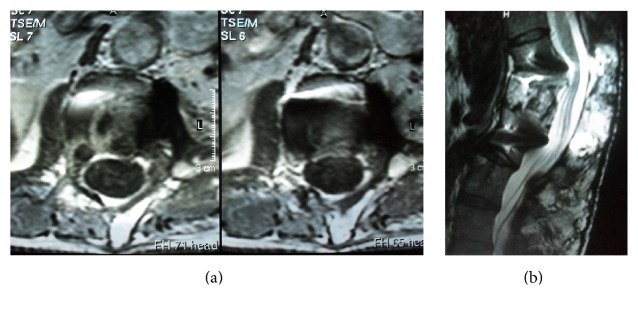
At the fifth year follow-up MRI, axial T_1_WI with gadolinium and sagittal T_2_WI show no recurrence.

At one-year follow-up, neurological status examination remained unchanged, but the CT scan showed that the tumor was more destructive (Figure 5(a)), while the MRI revealed that it was much larger and more invasive, with the dural sac now having been encircled and compressed (Figures 5(b) and 5(c)). A second more complete intra-arterial embolization procedure was performed (Figures 6(a), 6(b), and 6(c)). Total surgical removal of the hemangioma was achieved via an anterolateral approach that permitted the corpectomy of L1 and arthrodesis of Th12-L2 vertebrae by iliac graft and plaque (Figures 7(a) and 7(b)). The immediate postoperative MRI (Figures 8(a) and 8(b)) and again the fifth year (Figures 9(a) and 9(b)) and sixteenth year follow-up MRIs (Figures 8(c) and 8(d)) showed that the lesion was totally removed, fusion was stable, the hemangioma had not recurred, the spinal canal was free, and the kyphotic angle remained unchanged. The patient returned to his daily life and work with no complication and he was free of complaints.

## 2. Discussion

### 2.1. Characteristics

Vertebral hemangiomas are frequently encountered on plain radiographs, CT scans, and MRIs. These lesions are found in between 10 to 12% of routine autopsies but those which cause clinical signs are very rare; of spinal tumors treated, only between 2 and 3% are hemangiomas [1].

On plain radiographs and CT scans, vertically oriented vertebral lucencies separated by thickened trabecular bone are typical and give the appearance of honeycomb.

MRI shows the degree of soft tissue extension and the severity of the compression of the spinal cord. Vertebral hemangiomas can contain large feeding or draining vessels; hence, preoperative spinal angiography and preoperative embolization are highly recommended.

### 2.2. Pathogenesis

The formation of new blood vessels (neovascularization) is essential for normal tissue growth during embryonic development. Physiological neovascularization in adults is observed only in a few conditions such as wound and fracture healing, placenta formation, and the menstrual cycle. Abnormally activated neovascularization may occur in pathological states such as in cancer growth, diabetic retinopathy, and age-related macular degeneration.

A history of trauma has been elicited in up to 35% of the synovial hemangiomas of the knee. Also, in many patients with hemangioma of the skin, trauma has been regarded as an etiological factor [2, 3]. Recurrent trauma is thought to induce the growth of the hemangioma by mechanical irritation, which stimulates blood flow into preexisting lesions. Spinal microinstability and local angiogenic factor release causing the deviation of the angiogenetic pathways from normal healing process are other proposed pathogenesis theories in the development of posttraumatic hemangiomas but documented and published cases with an overt relationship are extremely rare [4–7].

### 2.3. Treatment

In the therapeutic armory, there are radiotherapy, intra-arterial embolization, direct ethanol injection, vertebroplasty, and surgery. They all have varying degrees of success. Surgical decompression is undertaken when significant or progressive neurological deficit is present.

One of the main causes of perioperative morbidity for patients undergoing surgical treatment for vertebral hemangioma is “excessive intraoperative blood loss” and postoperative hematoma [1]. Preoperative transarterial embolization has been found to reduce complications related to intraoperative blood loss and postoperative bleeding [1, 8–10].

Intra-arterial embolization followed by laminectomy is a safe and effective procedure for the treatment of cord compression by intraosseous vertebral body and posterior element hemangiomas. Gross total resection obviates the need for postoperative irradiation and minimizes the likelihood of recurrence [1].

Vertebral hemangiomas are radiosensitive lesions and doses as low as 30 to 40 Gy are effective [11, 12].

To our knowledge, this is the second report of posttraumatic extradural spinal hemangioma. The first case of extradural capillary hemangioma secondary to spinal trauma was published in 2011 by Shilton and involved an extradural homogeneously enhancing mass on a basis of T7 and T8 vertebral compression fractures following a motor vehicle accident ten years previously [6]. Another case of spinal cord capillary hemangioma with a history of spinal trauma ever to be published in English literature was mentioned by Abe, but in this case the lesion was intradural [13].

The present case demonstrates particularly the “growing” ability of the hemangiomas, as shown by the MRI from the first postoperative period. This growth potential obliged us to wait sixteen years before publishing our case; we believe that sixteen years of follow-up is a more than acceptable period of time in order to pronounce the absence of recurrence.

## 3. Conclusion

Although absolute proof of cause and effect as in our case is very rare, the emergence of a hemangioma in a fractured vertebra suggests that it is related to the deviation of the angiogenetic pathways from the normal healing process. An aggressive vertebral hemangioma that has been complicated with vertebra destruction and neural compression indicates the need for preoperative embolization and total surgical removal. Such a total resection can be safely achieved and seems to prevent recurrence without adjuvant therapy.
